# One‐Dimensional *π*–d Conjugated Coordination Polymer for Electrochromic Energy Storage Device with Exceptionally High Performance

**DOI:** 10.1002/advs.201903109

**Published:** 2020-09-15

**Authors:** Guofa Cai, Peng Cui, Wenxiong Shi, Samuel Morris, Shi Nee Lou, Jingwei Chen, Jing‐Hao Ciou, Vinod K Paidi, Kug‐Seung Lee, Shuzhou Li, Pooi See Lee

**Affiliations:** ^1^ School of Materials Science and Engineering Nanyang Technological University Singapore 639798 Singapore; ^2^ Key Laboratory for Special Functional Materials of Ministry of Education National & Local Joint Engineering Research Center for High‐efficiency Display and Lighting Technology School of Materials Science and Engineering, and Collaborative Innovation Center of Nano Functional Materials and Applications Henan University Kaifeng 475004 China; ^3^ Present address: School of Materials Science and Engineering State Key Laboratory of Separation Membranes and Membrane Processes Tiangong University Tianjin 300387 PR China; ^4^ Facility for Analysis Characterisation Testing & Simulation (FACTS) Nanyang Technological University Singapore 639798 Singapore; ^5^ Singapore‐HUJ Alliance for Research and Enterprise (SHARE) Nanomaterials for Energy and Water Nexus (NEW) Campus for Research Excellence and Technological Enterprise (CREATE) 1 Create Way Singapore 138602 Singapore; ^6^ Division of Environmental Science and Engineering Pohang University of Science and Technology 77 Cheongam‐Ro, Nam‐Gu Pohang Gyeongbuk 37673 Republic of Korea; ^7^ Beamline Research Division Pohang Accelerator Laboratory Pohang 790784 Republic of Korea

**Keywords:** conjugated coordination polymers, electrochromism, energy storage, indicators, smart windows

## Abstract

The rational design of previously unidentified materials that could realize excellent electrochemical‐controlled optical and charge storage properties simultaneously, are especially desirable and useful for fabricating smart multifunctional devices. Here, a facile synthesis of a 1D *π*–d conjugated coordination polymer (Ni‐BTA) is reported, consisting of metal (Ni)‐containing nodes and organic linkers (1,2,4,5‐benzenetetramine), which could be easily grown on various substrates via a scalable chemical bath deposition method. The resulting Ni‐BTA film exhibits superior performances for both electrochromic and energy storage functions, such as large optical modulation (61.3%), high coloration efficiency (223.6 cm^2^ C^−1^), and high gravimetric capacity (168.1 mAh g^−1^). In particular, the Ni‐BTA film can maintain its electrochemical recharge‐ability and electrochromic properties even after 10 000 electrochemical cycles demonstrating excellent durability. Moreover, a smart energy storage indicator is demonstrated in which the energy storage states can be visually recognized in real time. The excellent electrochromic and charge storage performances of Ni‐BTA films present a great promise for Ni‐BTA nanowires to be used as practical electrode materials in various applications such as electrochromic devices, energy storage cells, and multifunctional smart windows.

## Graphical abstract

A 1D *π*–d conjugated coordination polymer (CCP) is successfully fabricated in which specific organic linker and metal ions are selected with the aim of increasing the density of redox active centers and enabling conductive paths for charge transport. Moreover, this CCP enables dual electrochromic and energy storage functionalities in an integrated smart device.

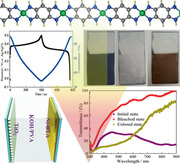

Owing to their aesthetic glazing, excellent dynamic control, as well as good coloration memory effect, electrochromic devices are emerging energy saving technologies for smart windows, displays, and antiglare automotive mirrors, etc.^[^ ^1^, ^2^, ^3^, ^4^ ^]^ Recent reports have shown that buildings installed with smart windows can offset as much as 40% energy consumption by reducing cooling, heating, and lighting needs.^[^ ^5^, ^6^ ^]^ Moreover, the dynamic coloration control afforded by electrochromic smart windows can protect users’ privacy and offer comfortable or aesthetic environment for occupants. To maximize the utilization of space in buildings, increasing research focus has been placed on integrating novel features and multi‐functionalities into electrochromic smart window.^[^ ^7^, ^8^, ^9^ ^]^ For example, several pioneer research works have been made to integrate energy storage and/or harvesting functionalities into the electrochromic smart window.^[^ ^10^, ^11^, ^12^ ^]^ However, further improvements in energy density, coulombic efficiency, cycling, and rate capability are essential for practical multifunctional smart windows. While both electrochromic and energy storage processes stem from electrochemical redox reactions, which are highly related to the surface area and electrical conductivity of the active materials, the development of high energy density electrochromic materials is governed by the need for large optical transmittance. Although metal oxide nanomaterials (such as NiO,^[^ ^13^ ^]^ V_2_O_5_,^[^ ^14^ ^]^ WO_3_,^[^ ^15^ ^]^ MoO_3_^[^ ^16^ ^]^) and conducting polymers (for instance, polyaniline)^[^ ^17^ ^]^ are widely investigated electrode materials for electrochromic devices to improve surface area or electrical conductivity, respectively, these properties cannot be achieved concurrently at present. Thus, there is a tremendous interest in developing new electrochemical active materials that exhibit both high surface area and electrical conductivity for more effective integration of electrochromic and energy storage properties. Meanwhile, band gap engineering has also been considered in the materials design for energy storage and electrochromic devices to tailor the capacity, coulombic efficiency, redox potential, stability, optical transparency, and so on.^[^ ^18^, ^19^, ^20^ ^]^

Coordination polymers (CPs), consisting of metal ions or clusters coordinated with organic linkers to form 1D (one dimensional), 2D, or 3D structures, are known for their high porosities, surface areas, tunable structures, and compositions.^[^ ^21^ ^]^ Benefiting from these exciting properties and versatility in structure designs at molecular level, CPs have been suggested as promising candidates for electrochromic or energy storage materials.^[^ ^22^, ^23^, ^24^, ^25^, ^26^, ^27^ ^]^ However, the poor electrical conductivities of most CPs significantly limit the effective utilization of built‐in redox centers, resulting in large polarization of redox peaks, small optical modulation, low capacity and rate capability, and poor electrochemical stability. Recently, two intrinsically conducting CPs have been designed and synthesized with excellent electrical conductivity and electrochemical stability in electrochemical double layer capacitor (EDLC) and pseudocapacitor.^[^ ^28^, ^29^ ^]^ In detail, moderate gravimetric capacity of about 30.8 mAh g^−1^ and volumetric capacity of about 32.8 mAh cm^−3^ were achieved by the EDLC,^[^ ^28^ ^]^ while higher gravimetric (59.3 mAh g^−1^) and volumetric (105.5 mAh cm^−3^) capacities can be realized by the pseudocapacitor.^[^ ^29^ ^]^ These energy storage performances are comparable or outperform most of the porous carbon electrodes, yet still inferior to state of the art energy storage materials. Moreover, there is no report on CPs that present electrochromic and energy storage properties simultaneously until now.

Ni‐based materials are of particular interest for delivering electrochemical activity, especially on energy storage and electrochromic fields. Moreover, Ni‐based CPs usually present high conductivities, for example, Ni_3_(2,3,6,7,10,11‐hexaiminotriphenylene)_2_ (Ni_3_(HITP)_2_) and Ni‐hexaaminobenzene (HAB) exhibit excellent conductivities of 5000 S m^−1[^ ^28^ ^]^ and 70 ± 15 S m^−1^,^[^ ^29^ ^]^ respectively. Based on these characteristics and advantages, we have synthesized a 1D *π*–d conjugated coordination polymer (CCP) with 1,2,4,5‐benzenetetramine (BTA) and nickel (Ni) as linkers and nodes, respectively. Interestingly, the synthesized Ni‐BTA nanowires could be vertically aligned on the substrates. The delocalization of electronic states on the 1D structure improves the conductivity of the coordination polymer. Additionally, 1D vertically aligned structure further facilitates the charge carrier transportation and increases the density of redox active centers because of the spaces among the nanowires. Different from previous CPs of which redox reactions are located on the organic linkers, the redox reaction of our 1D *π*–d CCP (Ni‐BTA) was centered on the Ni nodes, exhibiting exceptional electrochemical properties with applications being envisioned in the fields of electrochromic and energy storage. After growing onto transparent conductive FTO (fluorine doped tin oxide) glass by a facile and scalable chemical bath deposition (CBD) method, the constructed 1D *π*–d CCP based film presents excellent performance on both electrochromic and energy storage applications. All these performances are on par or surpass the state‐of‐the‐art energy storage devices based on conductive MOFs and carbon materials.

The BTA linker coordinates with *d* ^8^ metal species that prefer planar quadrilateral coordination geometry,^[^ ^30^ ^]^ which leads to the 1D *π*–d CCP structure (**Figure** **1**a). This ultimately yields ultrafine nanowires with diameter and length of ≈20 and 280 nm (Figure 1b,c), respectively. The Ni‐BTA nanowires could be uniformly grown on the whole surface of an FTO glass and almost vertically aligned to the substrate (Figure S1, Supporting Information). The thickness of the Ni‐BTA film is almost the same with the nanowire length (280 nm, inset of Figure 1b). Moreover, the 1D Ni‐BTA nanowires could be grown onto various substrates including nickel foam, carbon fiber, and textile (Figure S2, Supporting Information), indicating the universality of the synthesis route. The structure of Ni‐BTA nanowire was further investigated by transmission electron microscopy (TEM) image (Figure 1d). There are lattice‐resolved fringes with spacing between adjacent lattice planes of 0.215 nm in the high‐resolution TEM image of Ni‐BTA nanowire, which confirmed the crystallographic features of the 1D *π*–d CCP (inset of Figure 1d). X‐ray photoelectron spectroscopy (XPS) of the Ni‐BTA film reveals the presence of N, C, and Ni peaks in the sample while the O, Sn, and Si peaks stem from the FTO glass substrate (Figure S3a, Supporting Information). The high‐resolution Ni 2p spectra display peaks located at 854.4, 863.4, 871.7, and 880.7 eV (Figure S3b, Supporting Information) that can be attributed to the presence of Ni^2+^ in square planar geometry.^[^ ^31^ ^]^ The high‐resolution N 1s spectrum indicates a single type of N atoms (Figure S3c, Supporting Information). XPS analysis proved that the Ni‐BTA film is in a neutral state with absence of charge balance ions such as Cl^−^ and NH_4_^+^. Powder X‐ray diffraction (PXRD) measurement of Ni‐BTA displayed a semi‐crystalline structure with three prominent peaks at 2*θ* = 20.6°, 23.9°, and 29.3°, as well as some minor peaks at 2*θ* = 14.4°, 33.8°, 40.1°, 44.2°, and 60.1°, which correspond to the in‐plane periodicity and long‐range ordered stacking along *c*‐axis (Figure 1e). To verify the proposed 1D *π*–d conjugated molecular structure, density functional theory (DFT) calculations were conducted. An energetically favored P‐type 1D *π*–d conjugated coordination polymer with a P1 unit cell with parameters of a = 7.38 Å, b = 5.22 Å, c = 5.95 Å, *α* = 90.35°, *β* = 90.07°, and *γ* = 89.27° was constructed (Figure S4, Supporting Information). The unit cell parameters were refined against the PXRD data using a Pawley Fit in TOPAS 6. This yielded a *P1* cell of a = 7.388(5) Å, b = 5.253(7) Å, c = 6.055(8) Å, *α* = 91.0(2)°, *β* = 91.1(2)°, *γ* = 89.1(1)° with an *R*_wp_ = 1.92%, which is close to the DFT results, implying we have obtained the model structure.

**Figure 1:**
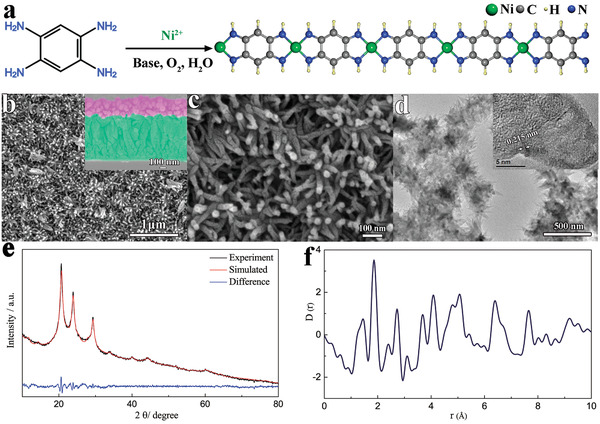
a) Synthesis scheme of Ni‐BTA. b) Top view and cross‐sectional SEM images of Ni‐BTA film on FTO glass (purple color, Ni‐BTA; green color, FTO layer). c) High magnification SEM images of Ni‐BTA nanowire. d) TEM images of Ni‐BTA nanowire. e) Experimental and simulated XRD patterns of Ni‐BTA nanowire powder. f) X‐ray pair distribution function (XPDF) of the Ni‐BTA nanowire powder.

While the unit cell could be refined against the PXRD, the lack of crystalline peaks inhibited a full structural Rietveld refinement. To supplement the structural characterization, we conducted X‐ray total scattering experiments at Diamond Light Source Synchrotron to further elucidate the real‐space atomic structure of the conjugated coordination polymer. An X‐ray pair distribution function (XPDF) of a Ni‐BTA sample was obtained by a Fourier transform of the corrected, weighted, total scattering diffraction. XPDF is a useful experimental technique for analyzing nanostructured materials as it takes into account both Bragg and diffuse scattering equally, allowing the user to investigate short and medium range orders of the sample.^[^ ^32^, ^33^ ^]^ The XPDF produced can be thought of as a histogram of interatomic distances within the sample, by using our DFT model, we were able to assign experimental peaks at ≈1.45, 1.86, 2.73, and 7.7 Å (Figure 1f), to C–C/C–N, Ni–N, Ni–C, and Ni–Ni pair correlations, respectively. At longer distances, the peaks cannot be easily assigned to single inter‐atomic distances, because of multiple overlapping contributions and correlations of similar distances. Additionally, the XPDF shows sharp peaks up to 10 Å indicating the presence of well‐ordered coordination units in our 1D *π*–d CCP. Moreover, no significant change in the XPDF was observed in 1D *π*–d CCP that was exposed to air for 2 months (Figure S5, Supporting Information), illustrating a good environmental stability of the 1D CCP that is resistant to atmospheric corrosion. Thermogravimetric analysis (TGA) measurement was performed for freshly cleaned Ni‐BTA powder (Figure S6, Supporting Information). The weight loss below 250 °C is attributed to dehydration of loosely bonded water, indicating the thermodynamic stability of Ni‐BTA up to 250 °C. The fast weight loss in the temperature range of 260–284 °C is assigned to the combustion of organic linkers. In the temperature range of 284–375 °C, the sluggish weight loss is attributed to a combination of weight gain from the oxidation of Ni nodes into NiO as well as the weight loss from combustion of organic linkers. When the temperature is higher than 400 °C, the organic linkers are completely removed and the Ni nodes are totally transformed to NiO. Moreover, the weight ratio of Ni in the TGA result is consistent with our proposed chemical formula (NiN_4_C_6_H_4_). The porosity of Ni‐BTA 1D CCP was investigated from the nitrogen sorption/desorption isotherms measured at 77 K (Figure S7, Supporting Information). The Brunauer–Emmett–Teller (BET) specific surface area was calculated to be 280.4 m² g^−1^. Ni‐BTA 1D *π*–d CCP exhibits a very steep desorption branch, which is a characteristic feature of H2 loops.^[^ ^34^ ^]^ The steep desorption branch can be attributed to pore‐blocking/percolation in a narrow range of pore necks, indicating a mesoporous structure, which is in good agreement with the pore size distribution in a narrow range from 2.5 to 4.7 nm with a pore volume of 0.3 cm³ g^−1^ (Figure S7b, Supporting Information).

Electronic structure and electrical conductivity of the Ni‐BTA 1D *π*–d CCP were further investigated. The electronic absorption spectroscopy shows a broad absorption from ultraviolet (UV) and visible (Vis) light to near infrared (NIR) region (**Figure** **2**a). The calculated optical bandgap of Ni‐BTA 1D *π*–d CCP is about 0.62 eV from the onset of the electronic absorption spectroscopy, which is consistent with the computational studies (Figure S8, Supporting Information). The narrow bandgap of Ni‐BTA 1D *π*–d CCP can be attributed to the strong interaction of the *π* orbitals on the radical anionic BTA and the d orbitals of Ni^2+^, which constructs the fully conjugated structure.^[^ ^35^ ^]^ The electrical conductivity was measured as a function of temperature through the Van Der Pauw method on a pressed pellet of Ni‐BTA nanowires (Figure 2b). To avoid the interruption of water or other solvents, the Ni‐BTA nanowires were dried in vacuum at 80 °C for 24 h before measurement. In addition, four tiny gold electrodes were sputtered on the four corners of the pellet for contact purpose (inset of Figure 2b). With numerous inter‐grain boundaries and nanometer dimensions, the Ni‐BTA nanowire based pellet still delivers an electrical conductivity of 1.1 × 10^−4^ S m^−1^ at room temperature. The measured electrical conductivity has a sharp enhancement with increasing temperature, indicating the semiconductive property of Ni‐BTA 1D *π*–d CCP. There is a 2180 times enhancement of the conductivity (2.4 × 10^−1^ S m^−1^) when the temperature reached 150 °C. Although the conductivity of Ni‐BTA 1D *π*–d CCP is lower compared to conductive metal organic frameworks (MOFs) such as Ni‐hexaaminobenzene (70 ± 15 S m^−1^)^[^ ^29^ ^]^ and Ni_3_(2,3,6,7,10,11‐hexaiminotriphenylen)_2_ (5000 S m^−1^),^[^ ^28^ ^]^ it is much higher than that of recently reported semiconductive MOF such as Cu‐hexahydroxybenzene (7.3 × 10^−6^ S m^−1^)^[^ ^36^ ^]^ and Cu[Ni(pyrazine‐2,3‐dithiolate)_2_] (1 × 10^−6^ S m^−1^).^[^ ^37^ ^]^ The high electrical conductivity of Ni‐BTA can be attributed to the delocalization of electrons in the *π*–d systems, in which nickel nodes can mediate efficient conjugation pathways between electroactive organic linkers.^[^ ^31^, ^35^, ^38^, ^39^ ^]^

**Figure 2:**
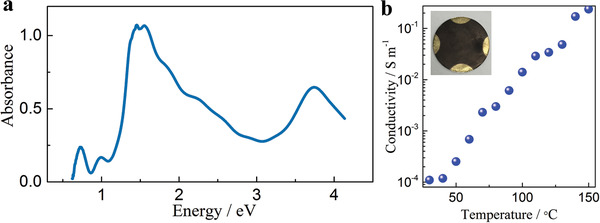
a) The electronic absorption spectroscopy of Ni‐BTA nanowires. b) Conductivity variation with respect to temperature plot. Inset: photograph of the Ni‐BTA nanowire pellet after sputtering gold electrodes symmetrically on the edges.

Encouraged by the large surface area and high conductivity of Ni‐BTA 1D *π*–d CCP, we envision that the Ni‐BTA nanowire film would possess superior electrochemical and electrochromic performance simultaneously. To validate the hypothesis, cyclic voltammetry (CV) was measured using a three‐electrode spectroelectrochemical cell (Ni‐BTA nanowires on conductive FTO substrate as a working electrode, Ag/AgCl as a reference electrode, Pt foil as a counter electrode, and conventional 1 m KOH aqueous solution as the electrolyte). The CV curves starting from first to sixth cycle at 5 and 10 mV s^−1^ were respectively performed to illustrate the initial electrochemical processes in detail (Figure S9, Supporting Information). It can be seen from the CV curves that the organic linkers and Ni nodes were oxidized at the first cycle (Figure S9a, Supporting Information). However, the oxidation reaction of the organic linkers is not fully reversible. After three scanning cycles at 5 mV s^−1^, Ni nodes could undergo reversible oxidation–reduction reactions, thus, the original blue color almost disappeared. Meanwhile, the redox peak intensities of Ni nodes increase with increasing scanning cycles, indicating an activating process for the Ni nodes (Figure S9b, Supporting Information). The original blue color totally disappeared after fifth cycle at 10 mV s^−1^, indicating the activation process of Ni nodes has been accomplished. Then, the Ni‐BTA nanowire film showed reversible color changes between brown color and transparent state, demonstrating the electrochromic processes occurred concurrently with the redox reaction. In order to investigate the electrochemical and electrochromic processes simultaneously, the CV at a scan rate of 10 mV s^−1^ and in situ dynamic transmittance spectra at 500 nm were measured (**Figure** **3**a). Over a potential range of 0 to 0.6 V versus Ag/AgCl, the Ni‐BTA nanowires exhibit one pair of pronounced redox peaks indicating a typical Faradaic behavior.^[^ ^40^ ^]^ The oxidative and reductive peaks at a scan rate of 10 mV s^−1^ are respectively located at 0.444 and 0.312 V versus Ag/AgCl, which are consistent with the Ni^2+^/Ni^3+^ redox couple observed in NiO and Ni(OH)_2_ films,^[^ ^41^, ^42^ ^]^ indicating the redox reaction was occurring at the nickel nodes of the Ni‐BTA 1D *π*–d CCP. In situ optical spectrum of the Ni‐BTA nanowire film revealed that the transmittance at 500 nm was dynamically reduced when the potential was increased from 0 to 0.6 V versus Ag/AgCl, and the transmittance was recovered to around the initial value when the potential was reversed back from 0.6 to 0 V versus Ag/AgCl (blue dot line in Figure 3a). Additionally, with increasing sweep rate from 5 to 100 mV s^−1^, the current subsequently increases and the voltage separation between oxidative and reductive peaks widens due to an increased internal resistance of the electrode. However, the sharp redox reaction peaks are still maintained even at a high scan rate of 100 mV s^−1^ (Figure S10, Supporting Information), suggesting a fast charge transfer and low internal resistance in the Ni‐BTA nanowire film. Moreover, the pronounced symmetric redox peaks illustrate an excellent electrochemical reversibility of the Ni‐BTA nanowire film.

**Figure 3:**
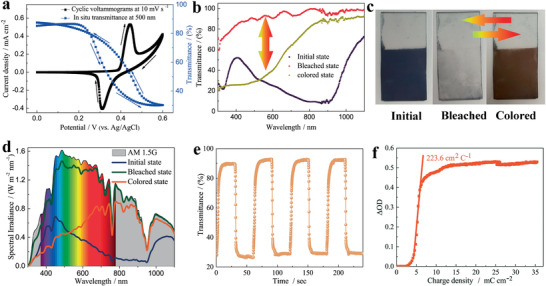
a) Cyclic voltammograms and in situ transmittance at 500 nm of Ni‐BTA nanowire film in 1 m KOH electrolyte at a scan rate of 10 mV s^−1^ in the potential range of 0–0.6 V versus Ag/AgCl. b) Transmittance spectra of Ni‐BTA nanowire film in the initial, bleached (0 V vs Ag/AgCl) and colored (0.6 V vs Ag/AgCl) states between the wavelength region of 300–1100 nm. c) Photographs of Ni‐BTA nanowire film in the initial, bleached, and colored states. d) Solar irradiance spectra converted from the measured transmittance spectra of Ni‐BTA nanowire film. e) In situ optical responses of the film with size of 4 cm^2^ under the applied potential square‐wave of 0 and 0.6 V versus Ag/AgCl for Ni‐BTA nanowire film for 60 s per step test at 500 nm. f) Optical density as a function of the charge density during coloring process at 500 nm.

We further investigated the electrochromic properties of the Ni‐BTA nanowire film by measuring the broadband transmittance spectra from 300 to 1100 nm for initial, colored, and bleached states, respectively (Figure 3b). The blue Ni‐BTA nanowire film in the initial state is opaque in the broad visible (Vis) light to near infrared (NIR) regions, hence blocking most of the transmittance in the wavelength region from 450 to 1000 nm. After applying a potential of 0.6 V versus Ag/AgCl on the Ni‐BTA nanowire film, the film became transparent in the NIR region, but it is still opaque (colored state) in the ultraviolet (UV) and most Vis light regions (from 300 to 680 nm). When the applied potential was changed to 0 V versus Ag/AgCl, the Ni‐BTA nanowire film turned transparent (bleached state) in the broad UV–Vis–NIR regions. Thus, this electrochemical reaction controlled state transformation could deliver an intriguing electrochromic phenomenon, with tunable transmittance covering the entire visible‐light and partial UV and NIR (three bands) regions. The optical modulation (Δ*T* = *T*_b_ − *T*_c_, where *T*_b_ and *T*_c_ denote the transmittance at the bleached and colored states, respectively) of 61.3% was observed at 500 nm, which is better or comparable than that of the conductive polymer thin films (<45%).^[^ ^43^, ^44^ ^]^ The uniform color distribution and obvious color changes were also recorded by capturing the digital photographs in each state (Figure 3c). We further studied the solar irradiation modulation of the Ni‐BTA nanowire film by converting the optical transmittance spectra to the solar irradiance transmittance in the 300–1100 nm region (Figure 3d). It was shown that our Ni‐BTA nanowire film can selectively modulate the dominating solar irradiance spectra. The Ni‐BTA nanowire film in the initial state blocks most of the solar energy in a broadband region. Note that 68.3%, 65.9%, and 90.4% of solar irradiance at 350, 500, and 900 nm were blocked by blue Ni‐BTA nanowire film. When the Ni‐BTA nanowire film was switched to the bleached state, it is almost transparent to the solar irradiance spectrum, only 24.98%, 10.01%, and 7.14% of solar irradiance at 350, 500, and 900 nm were blocked, respectively. On switching the Ni‐BTA nanowire film to the colored state, most of the solar irradiance in UV and Vis regions were blocked, but a large proportion of the solar heat in the NIR region can pass through the sample. At this state, 75.00% and 71.30% of the solar irradiance at 350 and 500 nm were blocked, while only 14.78% of solar heat at 900 nm was blocked. Undoubtedly, this phenomenon is very promising in smart window applications. For example, the initial state of the film with blue color can simultaneously protect the occupant's privacy and reduce energy consumption of the building by resisting heat transmission from summer heat. On the other hand, during the bleached state, the high transmittance of the film to solar irradiance permits both sunlight and solar heat transmission, which effectively reduce the energy consumption from the needs for heating and lighting in winter. The occupants can also selectively modulate the coloration intensity of the smart window to protect their privacy while conserving energy costs for lighting and heating. By applying a square‐wave potential between 0 and 0.6 V versus Ag/AgCl to the Ni‐BTA nanowire film, the corresponding alternating current (Figure S11, Supporting Information) and in situ transmittance spectra (Figure 3e) can be obtained. The switching speed (time required to reach 90% of its full optical modulation) can be calculated from the in situ transmittance spectrum, which is *t*_c_ = 1.8 s and *t*_b_ = 5 s for coloring and bleaching processes, respectively. The switching times are comparable with the MOF‐derived hierarchical‐porous NiO films,^[^ ^45^ ^]^ and much faster than that of the poly(3,3′‐dimethyl‐2,2′‐bithiophenyl) (PMe_2_T_2_)‐tin doped indium oxide (ITO) nanoparticles composites.^[^ ^44^ ^]^ Optical memory is one of the most important parameters for electrochromic material, which refers to the transmittance change at open circuit. To evaluate the optical memory of the Ni‐BTA nanowire film, the colored film was left in air without applied voltage and the transmittance was measured in real time (Figure S12, Supporting Information). The transmittance degrades <16% after 2000 s, indicating a satisfactory optical memory of the Ni‐BTA nanowire film. Coloration efficiency (CE), one of the key figure of merits of electrochromic materials, can be defined as the optical density (OD) changes per unit charge density (Q/A) inserted into/extracted out from the electrochromic materials during switching. CE can be calculated based on following equation:

(1) $$\begin{array}{c} \mathrm{CE}\left(\lambda \right)=\Delta \mathrm{OD}/\Delta Q/A=\log\left(T_{b}/T_{c}\right)/\Delta Q/A \end{array}$$

where T_b_ and T_c_ are the transmittance of the Ni‐BTA nanowire film in bleached and colored states, respectively. Therefore, the CEs calculated from the slope of the linear fit region in OD at 500 nm versus Q/A plots is 223.6 cm^2^ C^−1^ for the coloration process (Figure 3f). The CE value of coloration process is much higher than recently reported NiO^[^ ^46^ ^]^ and Ni(OH)_2_^[^ ^47^ ^]^ and most of the previous reported metal oxide electrochromic materials, which is usually <100 cm^2^ C^−1^ in visible range.^[^ ^48^, ^49^, ^50^, ^51^ ^]^ The transmittance changes versus cycle numbers at 500 nm were further measured to evaluate the electrochromic durability of Ni‐BTA nanowire film (Figure S13, Supporting Information). The Ni‐BTA nanowire film sustained the optical modulation of about 90.8%, 86.3%, and 67.9% of their initial value after subjected for 1000, 5000, and 10 000 cycles, respectively, illustrating the excellent electrochromic durability.

To elucidate the electrochromic mechanism, Raman and infrared spectra measurements of the Ni‐BTA nanowire film in different states were conducted (**Figure** **4**a,b). At initial state (blue color), two strong peaks located at 1461.1 and 1518.4 cm^−1^ in Raman spectra could be assigned to the skeleton vibration of benzene. The peaks at 1209.7 and 621.7 cm^−1^ correspond to the C‐N stretching mode and Ni‐N stretching mode, respectively, which are the characteristic peaks of the Ni‐BTA 1D *π*–d CCP (Figure 4a).^[^ ^52^ ^]^ The band at 1089.1 cm^−1^ is attributed to the FTO substrate. While applying a potential of 0.6 V versus Ag/AgCl, the film changed to brown color. At this state, two distinct peaks around 475.1 and 568.3 cm^−1^ appeared, which can be attributed to Ni^3+^ bond vibrations.^[^ ^53^ ^]^ The intensity of the skeleton vibration of benzene was reduced when the film was changed to colored state, and these characteristic peaks were also slightly blue shifted, which can be attributed to the transformations of benzenoid structure to quinonoid structure. In addition, when the potential was swept back to bleached state by applying 0 V (versus Ag/AgCl), the peaks at 621.7 and 1209.7 cm^−1^ corresponding to Ni^2+^‐N and associated C‐N stretching modes reappeared, respectively, while the peaks at 475.1 and 568.3 cm^−1^ corresponding to Ni^3+^ were reduced. The results imply Ni^2+^ was oxidized to Ni^3+^ on coloring and Ni^3+^ reduction to Ni^2+^ on bleaching processes, which is consistent with previously reported NiO^[^ ^53^ ^]^ and Ni(OH)_2_,^[^ ^47^ ^]^ thereby, suggesting that the electrochromic redox reaction mainly occurs on Ni nodes. The skeleton vibration peaks of benzene also recovered partly in the bleached state. The mechanism was further investigated by FTIR measurements (Figure 4b). For the fresh sample, the group of bands in the region of 1470–1540 cm^−1^ can be attributed to mixed C‐C stretching and C‐H and N‐H bending vibrations. The band located at 1580 cm^−1^ is related to the C=C stretching vibration in benzene ring. The band at 1336 cm^−1^ is ascribed to the stretching mode of N‐H. The strong peak around 1187 cm^−1^ is assigned to the C‐N stretching vibration. The band located at 716 cm^−1^ corresponds to the coordinated Ni‐N stretching vibration. The band at 812 cm^−1^ is a well‐known out‐of‐plane vibration in para disubstituted benzene. The peak at 1629 cm^−1^ is due to the bending mode of water molecules. After applying potential of 0.6 and 0 V versus Ag/AgCl to convert the film to colored and bleached states, respectively, the group of bands within the region of 1470–1540 cm^−1^ are changed to narrow bands centered at 1504 cm^−1^ and the band around 1580 cm^−1^ almost disappeared, indicating most of the benzenoid rings were transformed to quinonoid rings. Ni K‐edge X‐ray absorption spectroscopy measurements were also performed on the Ni‐BTA samples to identify the oxidation state and local structure of Ni nodes. The normalized X‐ray absorption near edge structure (XANES) spectra of Ni‐BTA nanowire film in the initial and 3rd cycle colored and bleached states are shown in Figure 4c. The XANES spectrum of the initial Ni‐BTA film presents a shoulder peak at 8340 eV, which can be ascribed to Ni(II) in a low spin square planar configuration,^[^ ^35^, ^54^ ^]^ as shown in Figure 1a. Interestingly, in the colored state, the absorption peak at 8340 eV is absent evidencing that the oxidation state of Ni is significantly different. Additionally, in the colored state, the intensity of the 8352 eV peak is increased compared to the initial Ni‐BTA sample suggesting that the Ni(II) nodes were oxidized to Ni(III). To further confirm the absorption peak at 8352 eV is attributed to Ni(III) oxidation state, the XANES spectrum of the third colored sample was compared with that of a Ni(III) standard (Ni_2_O_3_) (Figure 4d). The XANES spectrum of the third colored sample was similar to that of the Ni(III) standard. This indicates the Ni (II) nodes of the Ni‐BTA nanowires were oxidized to Ni^3+^ on coloration, which is consistent with the Raman (Figure 4a) and infrared spectra (Figure 4b). On bleaching, the XANES spectrum of the third bleached state shows a reappearance of the absorption peak at 8340 eV, which was ascribed to Ni(II) with a low spin square planar geometry as aforementioned, while concurrently, the absorption peak intensity at 8532 eV that is associated with Ni(III) decreased. This implies the Ni(III) nodes of the colored samples were reduced to Ni(II) on bleaching, which is also consistent with the findings from Raman (Figure 4a) and infrared (Figure 4b) spectra. Moreover, the Ni K‐edge XANES spectra of Ni‐BTA nanowire films after the eighth colored and bleached cycles (as shown in Figure S14, Supporting Information) demonstrate similar reversible changes in the Ni(II) and Ni(III) absorption peaks at 8340 and 8532 eV, respectively. On a whole, the partial restoration of the Ni(II) low spin square configuration on repeated bleaching and coloring indicate that the coloration and bleaching products of Ni‐BTA possess significant structural stability. We have also performed extended X‐ray absorption fine structure (EXAFS) measurements on the initial, colored, and bleached Ni‐BTA samples to further elucidate the local structure of Ni. The EXAFS spectrum of the initial Ni‐BTA sample modelled by P1 space group, as shown in Figure S15, Supporting Information, demonstrates four Ni‐N at ≈1.8 Å and four Ni‐C at ≈2.7 Å; which are in good agreement with the XRD, XPDF, and DFT results. The EXAFS fit parameters of the initial NI‐BTA sample are shown in Table S1, Supporting Information. It is important to note that while EXAFS measurements of the colored and bleached samples could provide Ni atomic distances situated in the Ni‐BTA colored and bleached products, we found that these EXAFS measurements offer low reliability owing to the gradual de‐colorization change of the colored samples on exposure to air during measurements.

**Figure 4:**
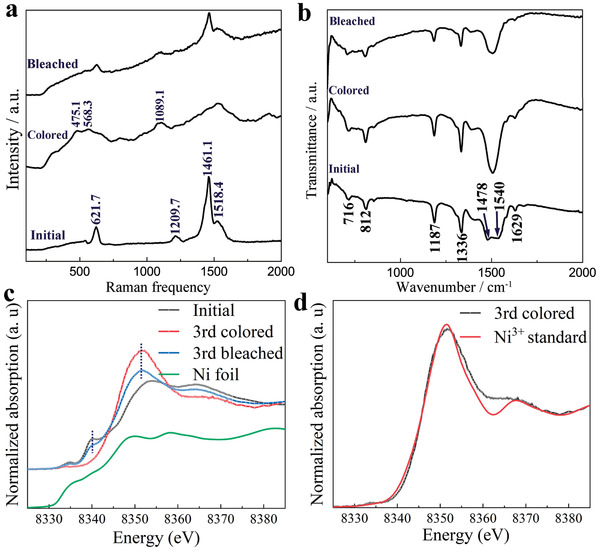
a) Raman spectra of Ni‐BTA nanowires in initial, colored, and bleached states. b) FTIR spectra of Ni‐BTA nanowires in initial, colored, and bleached states. c) Ni K edge XANES of Ni foil and Ni‐BTA nanowires in initial, third colored and bleached states and d) XANES of Ni‐BTA nanowires in third colored state presented with the Ni^3+^ standard (Ni_2_O_3_) as discussed in the text.

We further carried out quantitative analyses of the energy storage performance of the Ni‐BTA nanowire film on FTO glass in the same electrolyte showing battery‐like energy storage behaviors. Even on the transparent FTO substrate with moderate conductivity (about 10 Ω sq^−1^) and in the water based electrolyte (1 m KOH), the gravimetric and volumetric capacities of our Ni‐BTA film can achieve up to 168.1 mAh g^−1^ and 129.2 mAh cm^−3^ at current densities of 1.7 A g^−1^ and 1.28 A cm^−3^, respectively (**Figure** **5**a and Figure S16a, Supporting Information). The gravimetric capacity is 2.8 and 5.5 times higher than that of the recently reported conductive CCPs (30.8 and 59.3 mAh g^−1^ at current density of 0.05 A g^−1^ and scan rate of 0.2 mV s^−1^, respectively).^[^ ^28^, ^29^ ^]^ In addition, this Ni‐BTA nanowire film on FTO substrate exhibited an areal capacity of 3.36 µAh cm^−2^ at a current density of 0.033 mA cm^−2^ with a high transmittance of 90% at 500 nm (Figure S16b, Supporting Information). Moreover, Ni‐BTA nanowire film also presented excellent rate capability, high gravimetric (125 mAh g^−1^), volumetric (96.2 mAh cm^−3^), and areal capacities (2.5 µAh cm^−2^) could be retained even when the current density was increased 10 times to 16.7 A g^−1^, 12.8 A cm^−3^, and 0.3 mA cm^−2^, respectively. (Figure 5a,b and Figure S16, Supporting Information). Our Ni‐BTA nanowire film delivers a gravimetric energy density of 37.5 Wh kg^−1^ and a volumetric energy density of 28.8 mWh cm^−3^ at high power density of 5 kW kg^−1^ and 3.85 W cm^−3^, respectively (Figure S17, Supporting Information). These energy storage and electrochromic performances achieved by Ni‐BTA nanowire film are superior to other materials (Table S2, Supporting Information). During these tests, the energy storage and electrochromic behaviors of the Ni‐BTA nanowire film came from the same electrochemical redox reaction (operating within 0–0.6 V vs Ag/AgCl for electrochromic tests, 0–0.5V vs Ag/AgCl for energy storage tests) within the same electrolyte system. Therefore, it is very interesting to integrate both energy storage and electrochromic functions into a single device to develop an energy storage indicator from which the level of stored energy can be recognized visually by the color changes. In order to illustrate this interesting and useful concept, the charge–discharge curves and corresponding in situ transmittance at 500 nm were measured (Figure 5c). During the charging process, the Ni(II) nodes are oxidized to a higher valence state of Ni(III), the color of the film is changed from transparent to brown. When the Ni‐BTA nanowire film is charged to a potential of 0.5 V versus Ag/AgCl at a current density of 1.7 A g^−1^, it reached a fully charged state, the color of the Ni‐BTA nanowire film turns to dark brown. In the reverse process, the oxidized Ni(III) nodes are reduced Ni(II), which is accompanied by the fading brown color and the film recovered to transparent when the discharge process is completed at a potential of 0 V versus Ag/AgCl. One‐to‐one correspondence between the transmittance and the storage level was observed for the Ni‐BTA nanowire film. Therefore, different storage states of the device can be predictable by the dynamic noticeable color variation. Even at high current densities of 4.2 and 8.3 A g^−1^, sufficient color switching is achieved by the Ni‐BTA nanowire film on FTO glass (Figure S18, Supporting Information), illustrating the superior charge carrier transport capability of Ni‐BTA nanowire film. Moreover, in this single bi‐functional device, the consumed electrical energy of the electrochromic device can be fully reutilized, which is a promising strategy for energy savings. Furthermore, the energy storage and electrochromic performances are well preserved with electrochemical cycling test, in which almost 100% coulombic efficiency was kept up to 10 000 galvanostatic charge–discharge cycles; 89% and 51.4% of capacity was maintained after 1000 and 10 000 galvanostatic charge–discharge cycles measured at a relatively high current density (12.5 A g^−1^; Figure S19, Supporting Information). The optical modulation retains 66.4% at 500 nm when comparing the optical contrast before and after the 10 000 galvanostatic charge–discharge cycles (Figure 5d). In order to demonstrate the degradation mechanism of the Ni‐BTA nanowire film during the electrochemical cycling, the electrochemical impedance and IR drop during galvanostatic charge/discharge were measured (Figure S20, Supporting Information). The high‐frequency region of electrochemical impedance spectroscopy and IR drop in discharge curves are characteristics of internal resistance, which consists of the resistance of the electrode–electrolyte interface, the separator, and the electrical contacts. The low frequency region of electrochemical impedance spectrum is associated with the charge‐transfer resistance related to ion interfacial transfer, coupled with a double‐layer capacitance at the interface. Apparently, the internal resistance slightly increased after 1000 galvanostatic charge–discharge cycles, and then remained almost unchanged even upon extending the galvanostatic charge/discharge up to 10 000 cycles. However, the charge‐transfer resistance increased distinctly with increasing number of galvanostatic charge–discharge cycles. Therefore, we could conclude that the degradation of the Ni‐BTA nanowire film during the galvanostatic charge–discharge cycling is mainly caused by increased charge‐transfer resistance.

**Figure 5:**
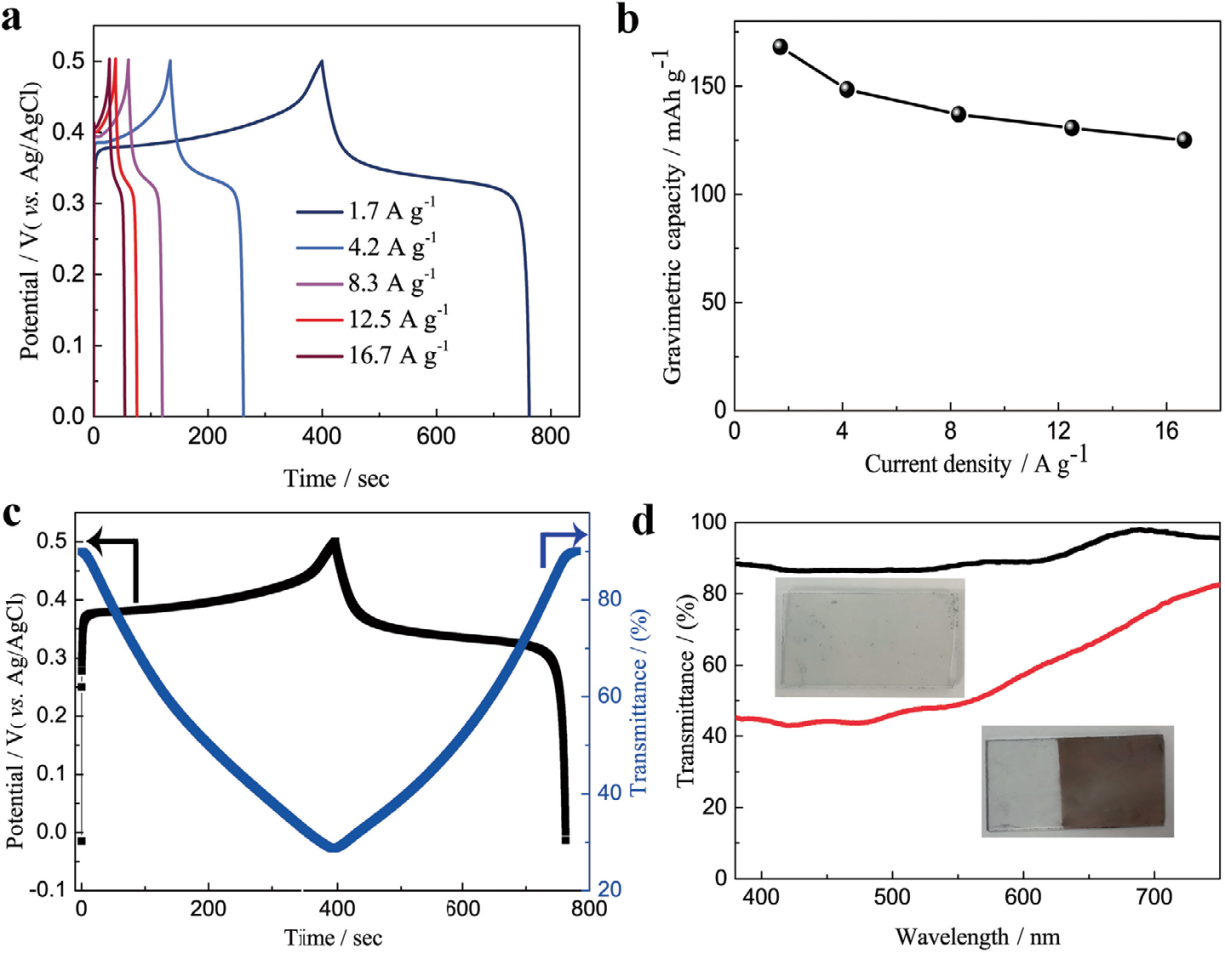
a) Galvanostatic charge/discharge profiles for Ni‐BTA nanowire film under different currents density in the potential range of 0–0.5 V versus Ag/AgCl (ranging from 1.7 to 16.7 A g^−1^). b) Gravimetric capacity of Ni‐BTA nanowire film as a function of the current density. c) Galvanostatic charge/discharge curve of Ni‐BTA nanowire film at a current density of 1.7 A g^−1^ and the corresponding optical responses spectra at 500 nm. d) Transmittance spectra of Ni‐BTA nanowire film after 10 000 charge–discharge cycles between 0 and 0.5 V (black line, bleached state; red line, colored state; inset: photographs of the Ni‐BTA film at bleached state and colored state, respectively).

We further explored a solid‐state electrochromic device based on Ni‐BTA nanowires film, sprayed TiO_2_ nanoparticles film, and KOH/polyvinyl alcohol (PVA) as the electrochromic layer, the ion storage layer, and the solid electrolyte, respectively (**Figure** **6**a). The solid‐state electrochromic device presented similar electrochromic process with the Ni‐BTA nanowires film as TiO_2_ is optical passive electrode material. After being activated by CV (Figure S21, Supporting Information), the solid‐state device exhibited high gravimetric and areal capacities of 125.2 mAh g^−1^ and 98 mAh m^−2^ (calculated based on the CV curve), respectively. In addition, our solid‐state device achieved large optical modulation in a broad visible light range and the value at 550 nm achieved up to 58% (Figure 6b). Moreover, fast switching speed (4 s /13.2 s, Figure 6d) and high CE (148 cm^2^ C^−1^, Figure 6e) were achieved for coloring and bleaching processes, respectively. Furthermore, the solid‐state device has a good bistability as the transmittance change <10% even open circuit for 2400 s at both colored and bleached states (Figure 6f). These superior performances are attributed to the vertically aligned superfine 1D nanowire array structure, high porosity, and the relative high conductivity of the Ni‐BTA nanowire as well as the good charge balance and storage capability of TiO_2_ in the device. The 1D nanowire array structure and porosity enable abundant active sites for electrolyte and ions. The intrinsic narrow bandgap of the Ni‐BTA nanowires provides sufficient conductive pathways for the electrons transport within the electrode.

**Figure 6:**
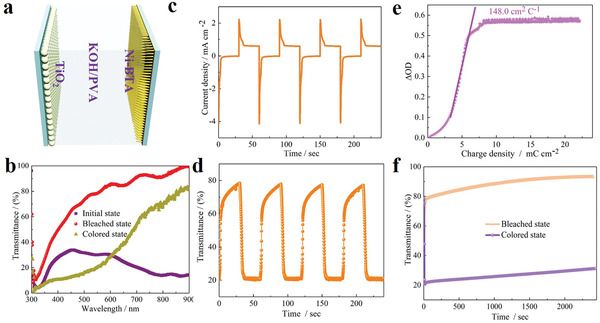
a) Configuration of the solid‐state device assembled by Ni‐BTA nanowires film as the electrochromic layer, sprayed TiO_2_ nanoparticles film as the ion storage layer, and KOH/PVA as the solid electrolyte. b) Transmittance spectra of the solid‐state device in the initial, bleached, and colored states between the wavelength region of 300 and 900 nm. c) Current response of the solid‐state device when applied the voltage range of between 0 and 1.5 V for 30 s per step. d) Corresponding in situ transmittance responses for 30 s per step measured at 500 nm. e) Optical density as a function of the charge density during coloring process at 500 nm. f) Transmittance changes at 500 nm of the solid‐state device in the colored and bleached states after power off for 2400 s.

We have developed a novel 1D CCP film based on the BTA organic linkers and Ni nodes via a facile CBD method. The CCP film exhibited an excellent electrochromic and energy storage performances even on transparent conductive FTO glass, including large optical modulation up to 61.3% at 500 nm, high CE (223.6 cm^2^ C^−1^), high gravimetric (168.1 mAh g^−1^) and volumetric (129.2 mAh cm^−3^) capacities as well as the long‐term electrochemical stability (>10 000 cycles) in aqueous electrolytes. Additionally, a smart energy storage indicator electrode was illustrated in which different storage states can be visually recognized in real time. To the best of our knowledge, this is the first report of CCP material, which synchronously exhibit both electrochromic and energy storage functions. Ultimately, a solid‐state device with excellent electrochromic and energy storage performance based on Ni‐BTA nanowires film, sprayed TiO_2_ nanoparticles film and KOH/ polyvinyl alcohol (PVA) respectively as the electrochromic layer, ion storage layer, the solid electrolyte was successfully assembled. Besides the electrochromic and energy storage applications, it is envisioned that this material will find broad applications in catalysis, sensing, and electronics.

## Conflict of Interest

The authors declare no conflict of Interest.

## Supporting information

Supporting Information
