# Supplementary material for: One‐Dimensional π–d Conjugated Coordination Polymer for Electrochromic Energy Storage Device with Exceptionally High Performance
Source: Adv Sci (Weinh). 2020 Sep 15;7(20):1903109. doi: 10.1002/advs.201903109 (PMC7578889; doi:10.1002/advs.201903109)
Supplement: Supplementary file 1 — Supporting Information [file ADVS-7-1903109-s001.pdf]

© 2020 Wiley-VCH GmbH

ADVANCED SCIENCE
Open Access

Supporting Information

for *Adv. Sci.*, DOI: 10.1002/advs. 201903109

One-Dimensional π–d Conjugated Coordination Polymer for Electrochromic Energy Storage Device with Exceptionally High Performance

*Guofa Cai, Peng Cui, Wenxiong Shi, Samuel Morris, Shi Nee Lou, Jingwei Chen, Jing-Hao Ciou, Vinod K Paidi, Kug-Seung Lee, Shuzhou Li, and Pooi See Lee**

Supporting Information

One-Dimensional π-d Conjugated Coordination Polymer for Electrochromic Energy Storage Device with Exceptionally High Performance

*Guofa Cai,[1,4,8] Peng Cui,[1,4,8] Wenxiong Shi,[1,5] Samuel Morris,[3] Shi Nee Lou, [1,2,6] Jingwei Chen, [1, 2] Jing-Hao Ciou,[1] Vinod K Paidi, [7] Kug-Seung Lee,[7] Shuzhou Li[1] and Pooi See Lee [1, 2]**

[1]School of Materials Science and Engineering, Nanyang Technological University, 639798, Singapore.

[2]Singapore-HUJ Alliance for Research and Enterprise (SHARE), Nanomaterials for Energy and Water Nexus (NEW), Campus for Research Excellence and Technological Enterprise (CREATE), 1 Create Way, Singapore 138602, Singapore.

[3]Facility for Analysis Characterisation Testing & Simulation (FACTS), Nanyang Technological University, 639798, Singapore.

[4]Key Laboratory for Special Functional Materials of Ministry of Education, National & Local Joint Engineering Research Center for High-efficiency Display and Lighting Technology, School of Materials Science and Engineering, and Collaborative Innovation Center of Nano Functional Materials and Applications, Henan University, Kaifeng 475004, China

[5]Present address: School of Materials Science and Engineering, State Key Laboratory of Separation Membranes and Membrane Processes, Tianjin Polytechnic University, Tianjin 300387, PR China.

[6]Division of Environmental Science and Engineering, Pohang University of Science and Technology, 77 Cheongam-Ro, Nam-Gu, Pohang, Gyeongbuk, Republic of Korea 37673

[7]Beamline Research Division, Pohang Accelerator Laboratory, Pohang 790-784, Republic of Korea

[8]These authors contributed equally to this work.

**E-mail: pslee@ntu.edu.sg*

Methods

Materials. 1,2,4,5-benzenetetramine tetrahydrochloride (BTA·4HCl, technical grade), nickel chloride hexahydrate ($NiCl_2·6H_2O$, 99.9%), potassium hydroxide (KOH, 99.95%) and concentrated aqueous ammonia (NH_4OH, ACS reagent, 28%-30% NH_3) were purchased from Sigma-Aldrich. Fluorine-doped tin oxide (FTO)-coated transparent conductive glass was purchased from Zhuhai Kaivo Electronic Components Co., Ltd. All the chemicals were used as received. Deionized (DI) water (Milli-Q 18 MΩ, Millipore Corp.) was used for all experiments.

Typical synthesis of Ni-BTA film by CBD route. The pre-cleaned FTO glass was used as the transparent conductive substrate and the nonconductive side was covered with polyimide tape to prevent Ni-BTA deposition on this side. The substrate was vertically supported on the wall of an open bath container. The solution for CBD reaction was prepared by mixing a solution of 484.2 mg (2.04 mmol) of $NiCl_2·6H_2O$ in 30 ml of DI water and a solution of 384 mg (1.35 mmol) of BTA·4HCl in 210 ml of water. Thereafter, 6.9 ml of concentrated NH_4OH was added to the mixture under a vigorous stirring. The reaction continued for 4 h under stirring at ambient conditions. Finally, the Ni-BTA films were obtained by removing the polyimide tape, and then washed with DI water, ethyl alcohol, respectively, and dried under room temperature for 6 h.

Solid-state device assembly. The solid-state electrochromic device was assembled by employing Ni-BTA nanowires film as the electrochromic layer, sprayed TiO_2 nanoparticles film as the ion storage layer, 1M KOH/ polyvinyl alcohol (PVA, 10wt%) as the solid electrolyte and VHB clear mounting tape (4010, 3 M) as the

spacer, respectively. Ultimately, the solid-state electrochromic was encapsulated *via* epoxy.

Sample Characterization. The crystalline structure of the Ni-BTA powder was investigated by X-ray diffraction (XRD, Bruker D8 Advance) technique with Cu-Kα-radiation (λ=1.541874 Å). TOPAS 6 was used to undergo Pawley fit on the PXRD data. In order to maintain a stable refinement and to minimise parameter correlation, the unit cell, background and peak shape parameters were refined separately. Microstructure and morphology of Ni-BTA nanowires film on FTO glass were observed with a field emission-scanning electron microscope (FESEM, JEOL 7600F) at 5.0 kV and an atomic force microscope (AFM, Asylum Research). Transmission electron microscopy (TEM) was performed on a JEOL JEM 2010 microscope operated at 200 kV accelerating voltage to observe the genuine microstructural informantion. X-ray pair distribution function (XPDF) data was collected at the I15-1 beamline at the Diamond Light Source, UK (λ = 0.161669 Å). Samples with small amount for the XPDF was loaded into a glass capillary with a diameter of 0.76 mm. Data on the sample, empty capillary and instrument data were collected. Background, container scattering, compton scattering, multiple scattering and absorption corrections were processed with the GudrunX program to achieve a Q = 22 Å^{-1}.[s1, s2] Thanks go to the beamline staff for completing the work as part of a rapid access call EE. X-ray absorption fine structure (XAFS) measurements were performed at 8C nano-probe XAFS beamline (BL8C) of Pohang Light Source (PLS-II) in the 3.0 GeV storage ring, with a ring current of 250 mA. The X-ray beam was monochromated by a Si(111) double crystal where the

beam intensity was reduced by 20% to eliminate the higher-order harmonics. The x-ray beam was then delivered to a secondary source aperture where the beam size was adjusted to be 0.5 mm (v) × 1 mm (h). The Ni K edge XAFS measurements on the initial, colored, and bleached thin films were measured at room temperature in fluorescence mode with standard 45° geometry and calibration was done using Ni foil. Due to the low concentration of Ni a four element Si drift detector was used to monitor the fluorescence x-rays and dead time corrections were taken into account. Typically, 3–4 scans were averaged for an improved signal-to-noise ratio of the initial and bleached samples, however, because colored samples are prone to oxidization (over time) only one scan was obtained immediately after electrochemical cycling. All the obtained spectra were processed using Demeter[1] package. Keeping the signal to noise ratio of the data in mind extended x-ray absorption fine structure (EXAFS) analysis was only performed on the initial system (Ni-BTA) with Fourier-transform range of 3.5 – 12 $Å^{-1}$ using a Hanning window applied between 1.2 Å and 2.7 Å. The amplitude reduction factor (S_o^2) was obtained by fitting the Ni metal. Photoelectron spectroscopy (XPS) was carried out on PHI Quantara II Scan X-Ray Microscope with monochromatic Al Kα irradiation (1486.6 eV, beam size is 100 μm in diameter). To confirm the electrochromic and energy storage reaction mechanism, Raman and fourier transform infrared spectroscopy (FTIR) were performed using a confocal Raman spectroscopy at 488 nm laser line (WITec, alpha300 SR) and a GX FTIR spectrometer (PerkinElmer Inc., Waltham, MA, USA), respectively. Gas-adsorption measurements were conducted on a Tristar II 3020 analyzer at 77 K.

The specific surface area and pore volume were analyzed through a Brunauer-Emmett-Teller (BET) using N_2 gas and Barrett-Joyner-Halenda (BJH) analysis methods, respectively. Thermogravimetric analyses (TGA) was measured by a TA Q500 system.

Electrochemical characterization. The electrochemical and electrochromic measurements were performed using a three-electrode electrochemical configuration and 1 M KOH aqueous solution as the electrolyte. The Ni-BTA nanowires film on FTO glass served as the working electrode, Ag/AgCl worked as the reference electrode and a Pt foil with a size of 2.5×4 cm^{-2} was used as the counter electrode. Cyclic voltammetry (CV), square-wave potential and galvanostatic charge–discharge measurements were carried out on an Solartron 1470E. CV curves with different scanning rates were measured between 0 V and 0.6 V vs. Ag/AgCl. The galvanostatic charge–discharge profiles with different current densities were measured from 0 to 0.5 V vs. Ag/AgCl. In situ electrochromic performance of the Ni-BTA nanowire films in response to different electrochemical conditions (CV, potential square-wave and galvanostatic charge–discharge) were evaluated through the combination of a UV–vis spectrophotometer (SHIMADZU UV-3600) and Solartron 1470E. The transmission spectra of the Ni-BTA nanowire films over the wavelength range from 300 to 900 nm in the bleached and colored states were measured, respectively. The electrochemical impedance spectroscopy (EIS) measurements were carried on in the frequency range of 100kHz to 0.01Hz under open circuit voltage. The gravimetric capacity (C_g), and

volumetric capacity (C_v) and areal capacity (C_a) of Ni-BTA films on FTO substrates are calculated according to the following equation.

$$C_g = I\Delta t/3600M \quad (1)$$

$$C_v = I\Delta t/3600V \quad (2)$$

$$C_a = I\Delta t/3600A \quad (3)$$

Where I (mA) and Δt (sec) denote the discharge current and time, respectively. M (g), V (cm^3) and A (cm^2) are the weight, volume and area of the Ni-BTA film, respectively. Typically, the loading mass of the Ni-BTA film used in the experiment is 0.02 mg cm^{-2}.

Molecular Dynamics (MD) simulation. MD simulations were conducted on the radial distribution function (RDF) of molecules using the GROMACS 4.07 simulation package[s3] and GROMOS96 force fields.[s4] The detail simulation information of force filed parameters of molecules, the structure information of simulation system, and simulation conditions were described in the following. The structures of molecules were obtained from DFT calculation within unit cells. The cutoff distance of 12 Å was utilized for short-range non-bonded interactions and the PME approach was used for long-range electrostatic forces. The system was subjected to a steepest descent energy-minimization, then to further thermalization at 300 K by NVT ensemble.

DOS Calculations of Geometric and Electronic Structures. The geometric and electronic structures calculations of molecules were calculated at the density functional theory (DFT) plane-wave level utilizing the Vienna ab initio simulation package (VASP)[s5, s6] with the projected augmented wave method (PAW).[s7] The

generalized gradient approximation (GGA) was used in these calculations. The calculations were carried out with the (9 × 9 × 9) Monkhorst-Pack k-points using a 400 eV cutoff energy for the molecules.

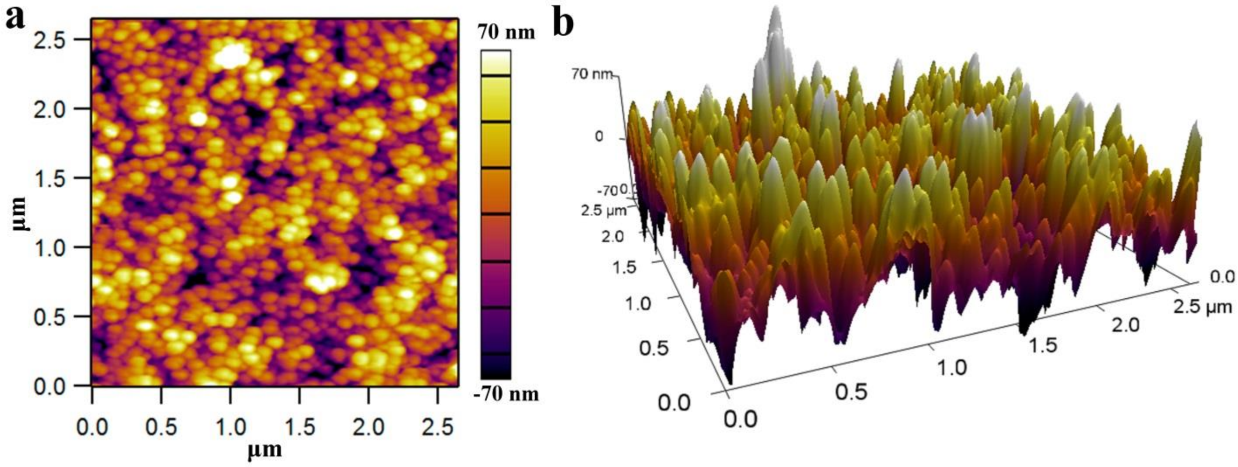

Figure S1 (a) Top view and (b) corresponding 3D AFM image of the Ni-BTA nanowires film on FTO glass.

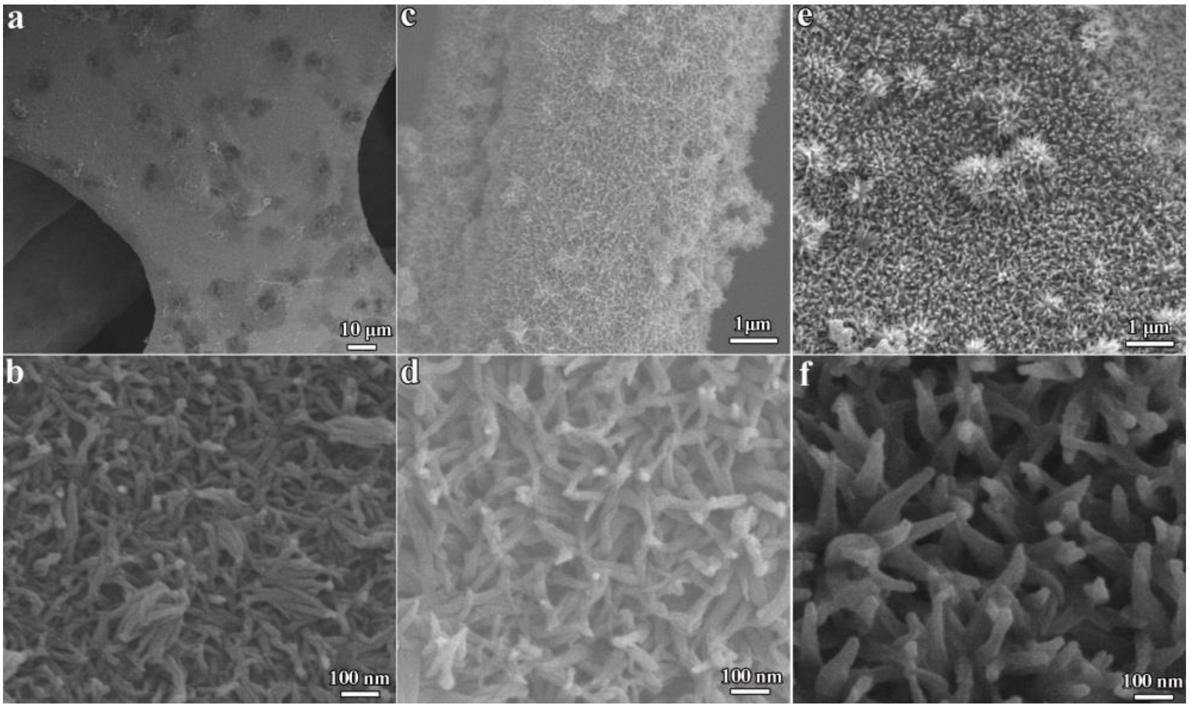

Figure S2 SEM images of the Ni-BTA nanowires film on different substrates. (a, b) Nickel foam, (c, d) carbon fibre and (e, f) normal cloth.

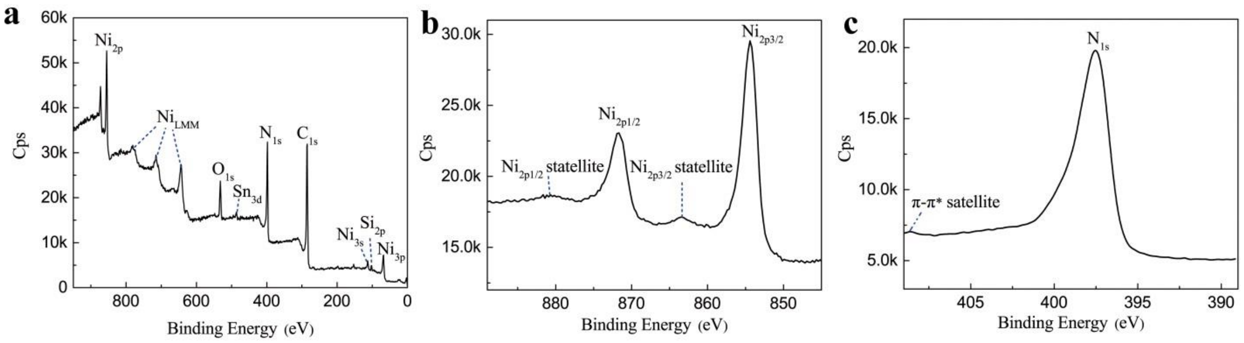

Figure S3 XPS spectra of the Ni-BTA nanowires film on FTO glass. (a) Survey spectra, (b) narrow scans Ni_{2p} core-level spectra, and (c) highly resolved N_{1s} spectra.

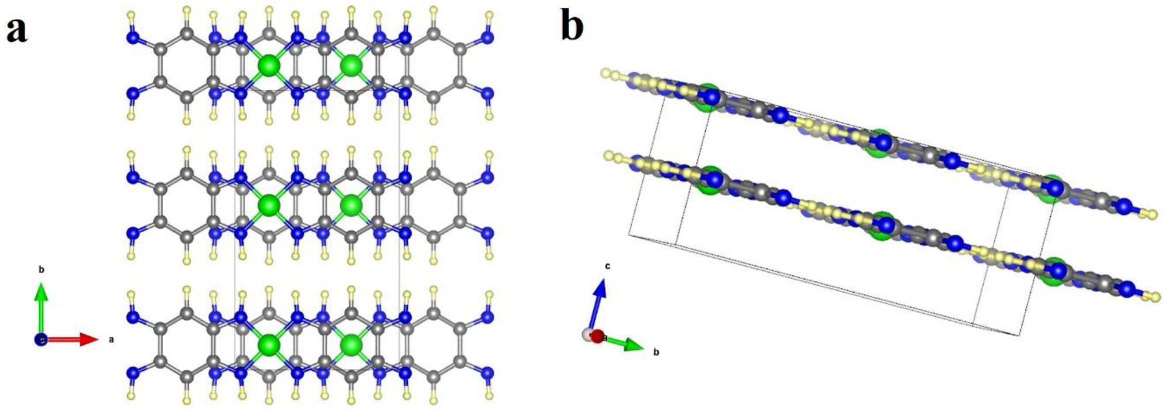

Figure S4 (a) Top and (b) side view of the simulated structure information of Ni-BTA.

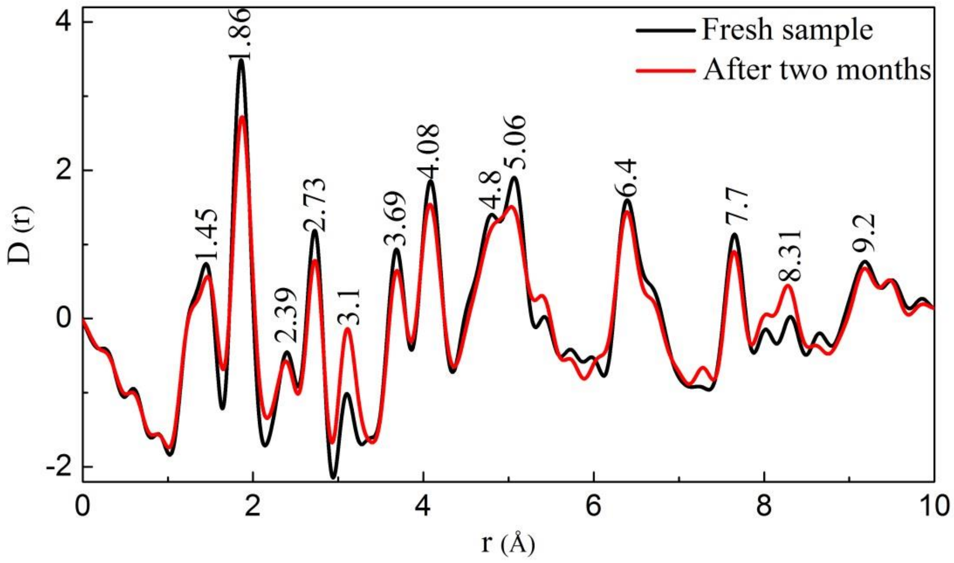

Figure S5 X-ray pair distribution function (XPDF) for fresh Ni-BTA nanowires and the sample exposed in air for 2 months.

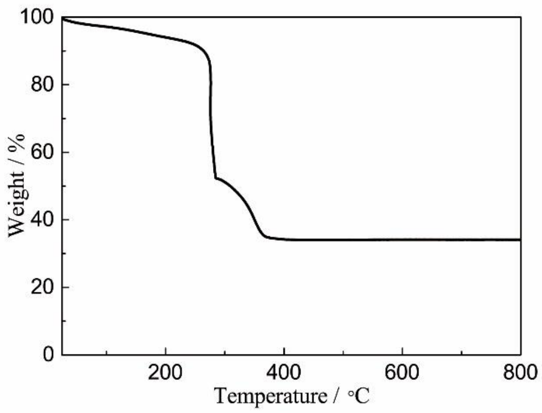

Figure S6 TGA curve of the Ni-BTA nanowires.

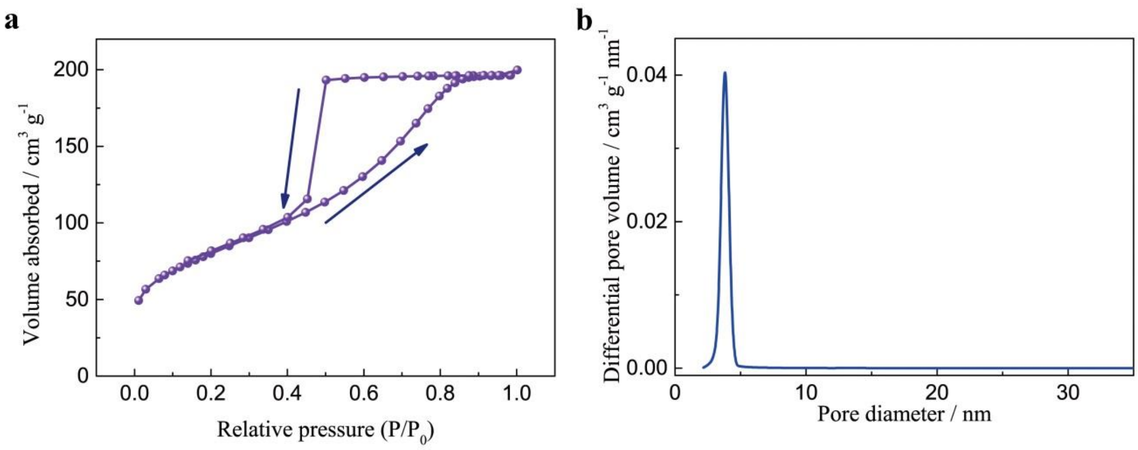

Figure S7 (a) N_2 adsorption isotherm and (b) the pore size distribution of the Ni-BTA nanowires.

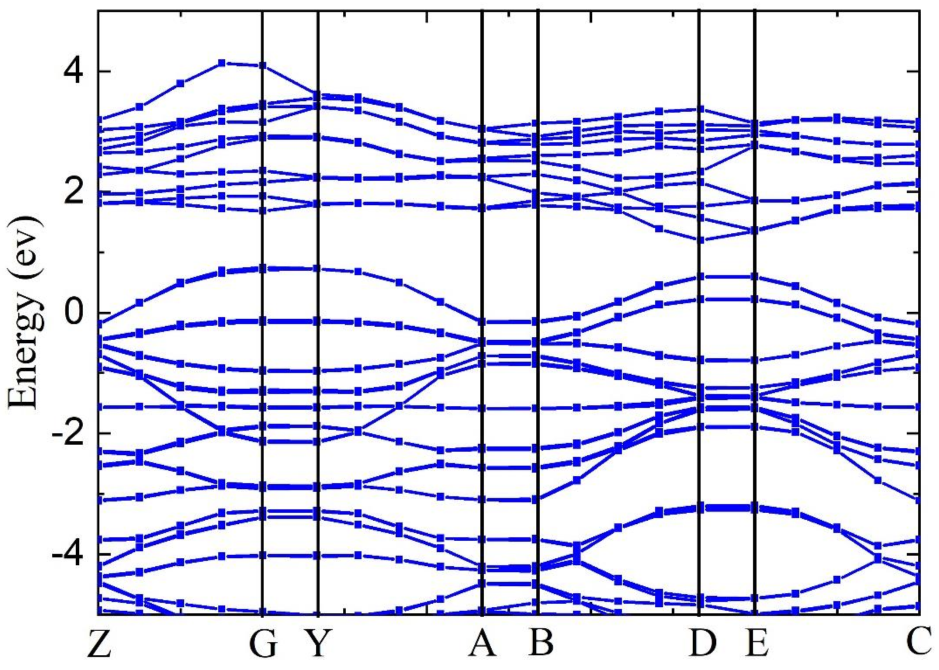

Figure S8 The simulated band structure of Ni-BTA.

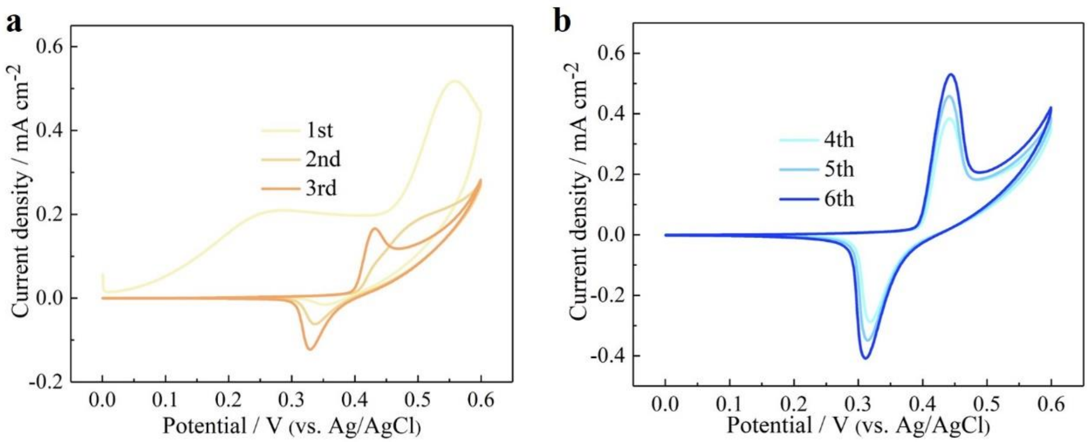

Figure S9 The 1st to 6th cyclic voltammograms of Ni-BTA nanowires film in 1 M KOH electrolyte at (a) 5 mV s^{-1} and (b) 10 mV s^{-1} in the potential range of 0–0.6 V vs. Ag/AgCl.

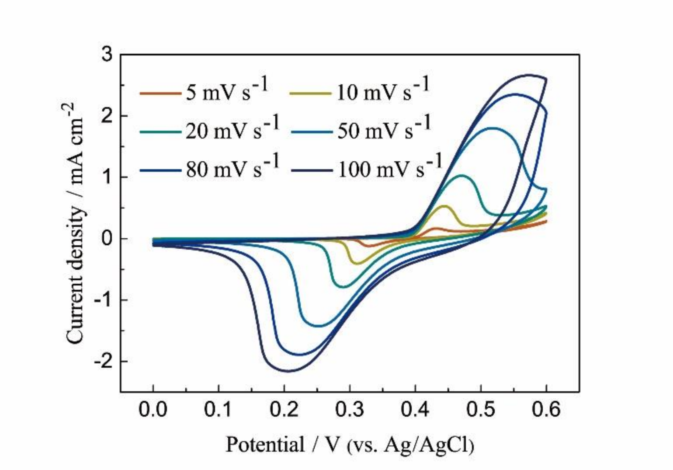

Figure S10 Cyclic voltammograms of Ni-BTA nanowires film in 1 M KOH electrolyte at different scan rates from 5 to 100 mV s^{-1} (after activating three times in each scan rate) in the potential range of 0–0.6 V vs. Ag/AgCl.

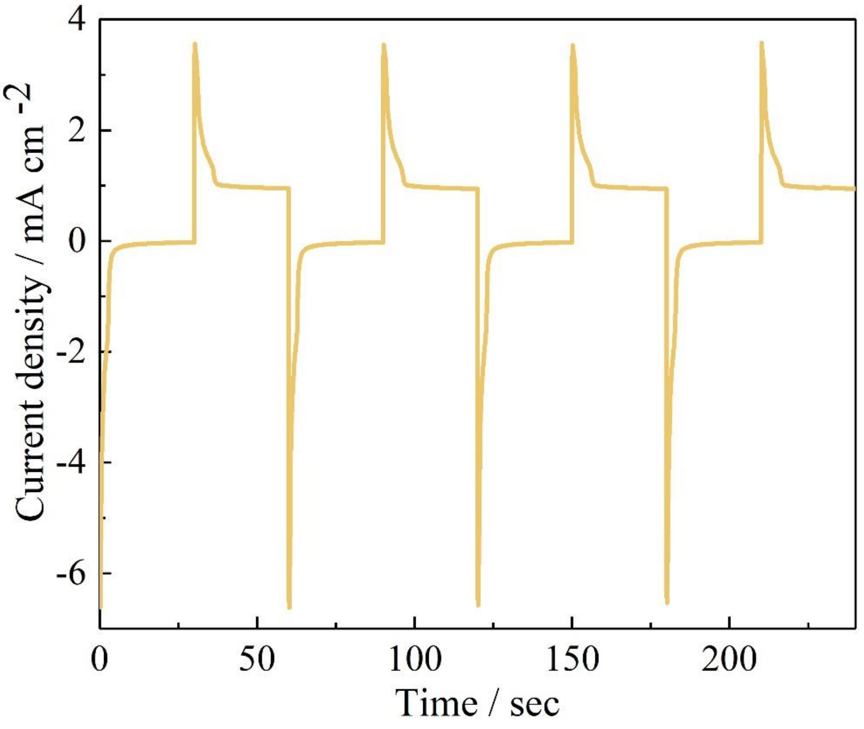

Figure S11. Corresponding current response of the Ni-BTA nanowires film on FTO glass when applied switching potential between 0 and 0.6 V vs. Ag/AgCl.

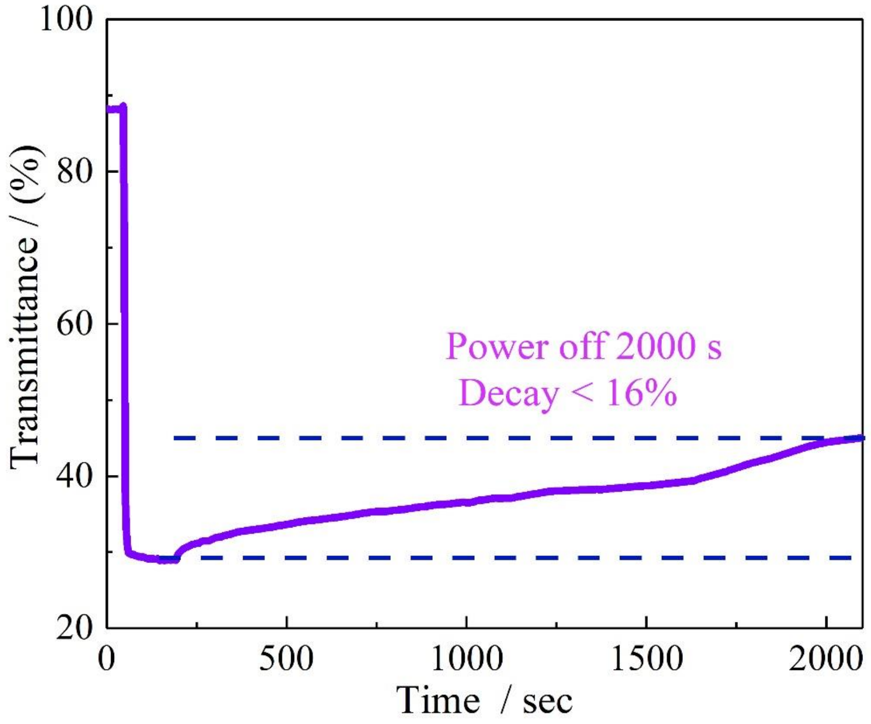

Figure S12 The transmittance change of the Ni-BTA nanowires film at 500 nm after being colored and then turning off the power (under open circuit).

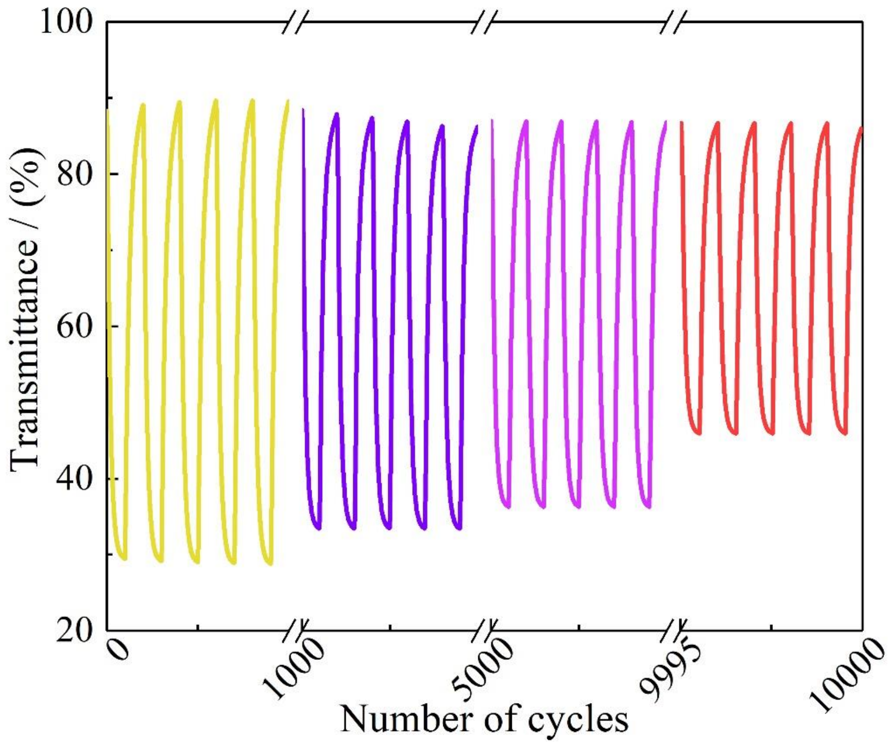

Figure S13 Cycle performance of the Ni-BTA nanowires film measured in 1M KOH for 10000 cycles.

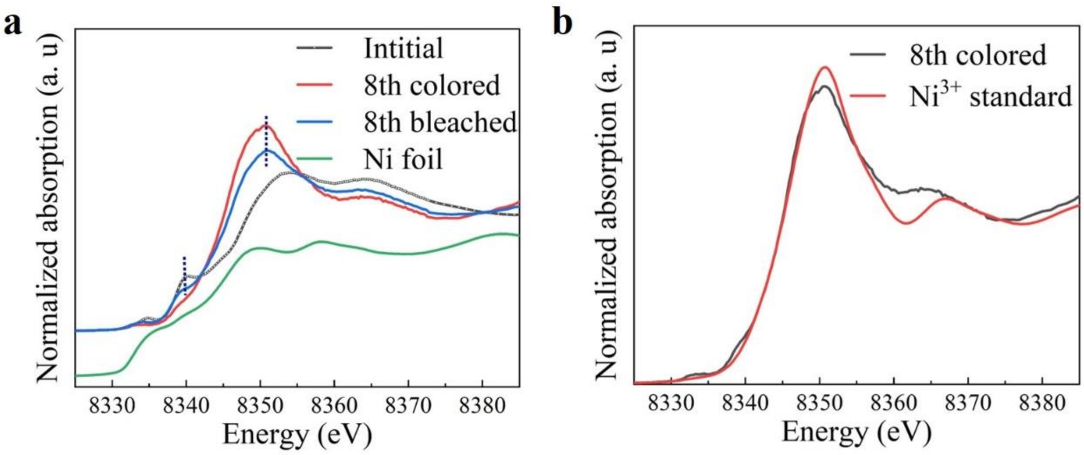

Figure S14 a) Ni Kedge XANES of Ni foil and Ni-BTA nanowires in initial, 8th colored and bleached states and b) XANES of Ni-BTA nanowires in 8th colored state presented with the Ni3+ standared.

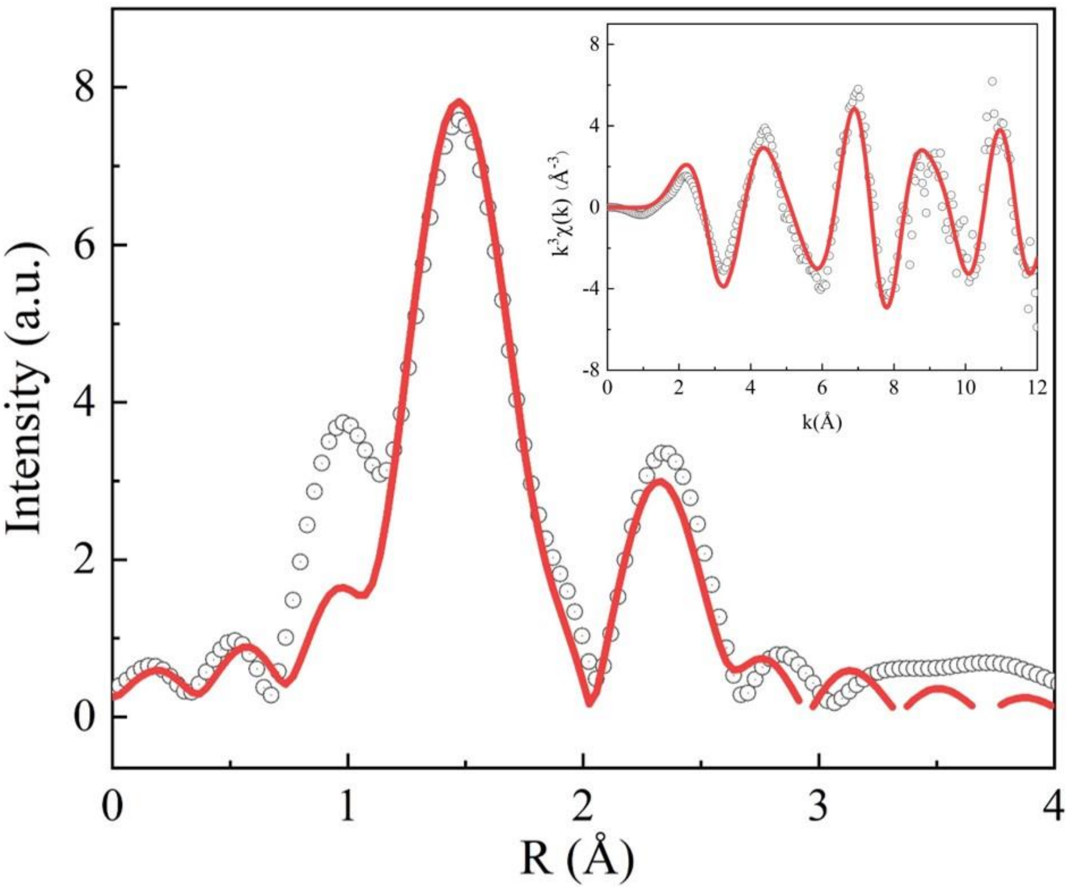

Figure S15 The Fourier transformed EXAFS data (open circles) and fit (solid line) is shown. EXAFS oscillations (k3 χ(k)) are shown in the inset. The Fourier transforms represent raw data without correcting for phase shifts.

Table S1: The results obtained from the fit parameters are presented in the table, here N = coordination number, E_0 = energy shift, R = interatomic distance, σ^2 = Debye-Waller factor (bond disorder), and R-factor = a measure of the quality of EXASFS fit

**Path**	**N**	**E_0 (eV)**	**R (Å)**	**σ^2 ($Å^2$)**	**R-factor**
Ni – N	4	-1(2)	1.87(1)	0.0018(5)	0.0017
Ni - C	4	-1(2)	2.72(2)	0.0018(5)	

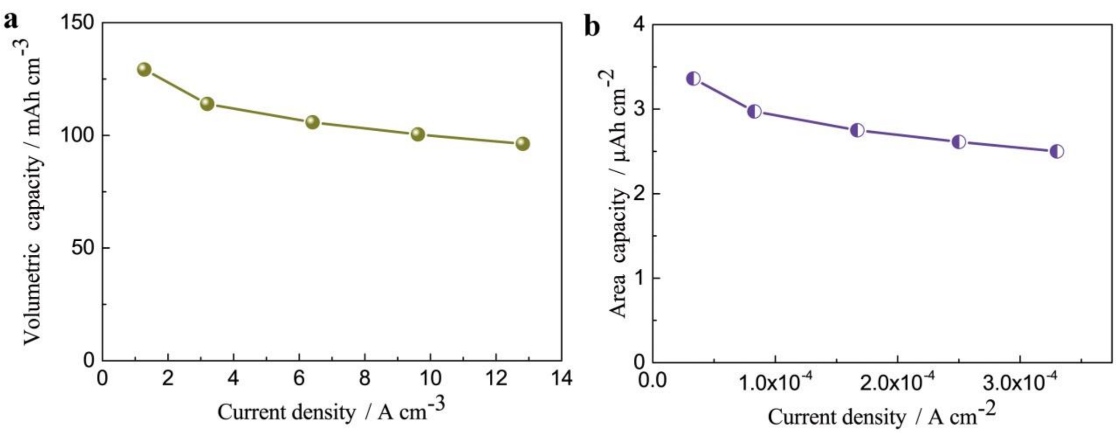

Figure S16 (a) Volumetric and (b) areal capacities of Ni-BTA nanowires film as a function of the current density.

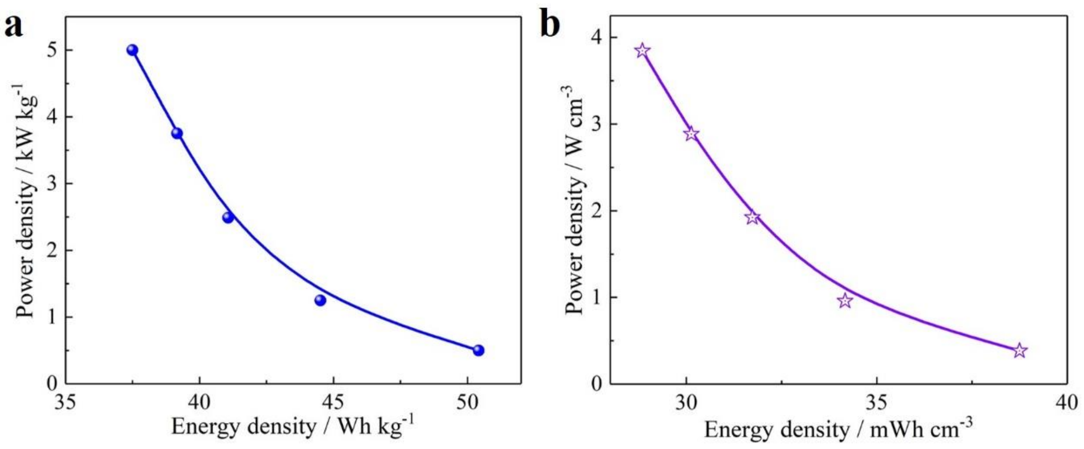

Figure S17 (a) gravimetric and (b) volumetric Ragone plot (power density vs. energy density) of the Ni-BTA nanowires film.

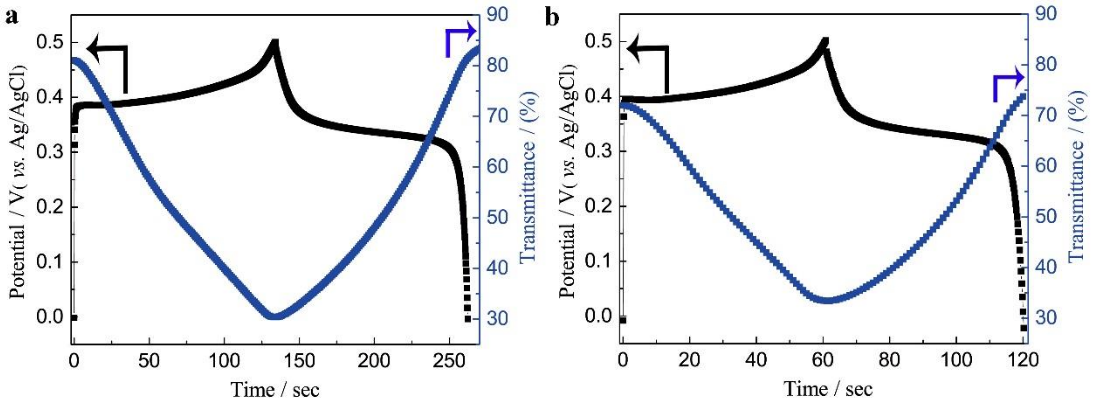

Figure S18 Galvanostatic charge/discharge curve of Ni-BTA nanowires film on FTO glass at different current densities and the corresponding optical responses spectra at 500 nm (a) 4.2 A g^{-1}, (b) 8.3 A g^{-1}.

Table S2. The performance of the state-of-the-art electrochromic and energy storage materials.

Material	Electrochromic performance			Energy storage performance			Ref.
	ΔT%	t_b/t_c (s)	CE (cm^2 C^{-1})	Capacity(max)	E_{max}	P_{max}	
WO_3 nanowires	43%	2.6/2.5	135.5	75 mAh g^{-1}	Non.	Non.	S8
MoO_3-$W_{0.71}Mo_{0.29}O_3$	46.7%	>20	20.8	41.9 mAh g^{-1}	Non.	Non.	S9
NiO	63.6%	9.5/11.5	42.8	42.7 mAh g^{-1}	10.6 Wh kg^{-1}	0.5 kW kg^{-1}	S10
$Ni_3(HITP)_2$	Non.	Non.	Non.	30.8 mAh g^{-1} 32.8 mAh cm^{-3}	15.4 Wh kg^{-1}	Non.	S11
Ni(HAB)	Non.	Non.	Non.	59.3 mAh g^{-1} 105.5 mAh cm^{-3}	14.8 Wh kg^{-1} 26.4 mWh cm^{-3}	3.05 kW kg^{-1} 5.43 W cm^{-3}	S12
Ni-BTA	58.3%	5/1.8	179.7	168.1 mAh g^{-1} 129.2 mAh cm^{-3}	50.4 Wh kg^{-1} 38.75 mWh cm^{-3}	5 kW kg^{-1} 3.85 W cm^{-3}	**This work**

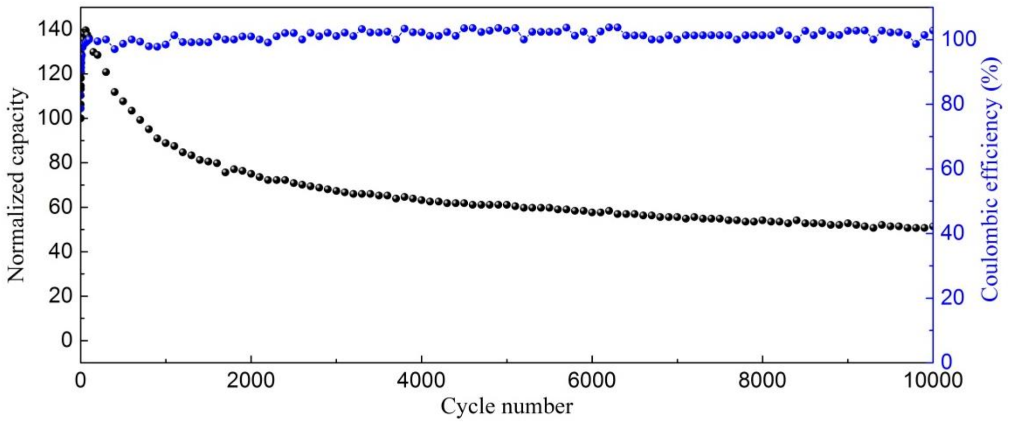

Figure S19 Electrochemical stability and coulombic efficiency of Ni-BTA nanowires film on FTO glass during a long term cycling of 10000 galvanostatic charge/discharge cycles at a current density of 12.5 A g^{-1}.

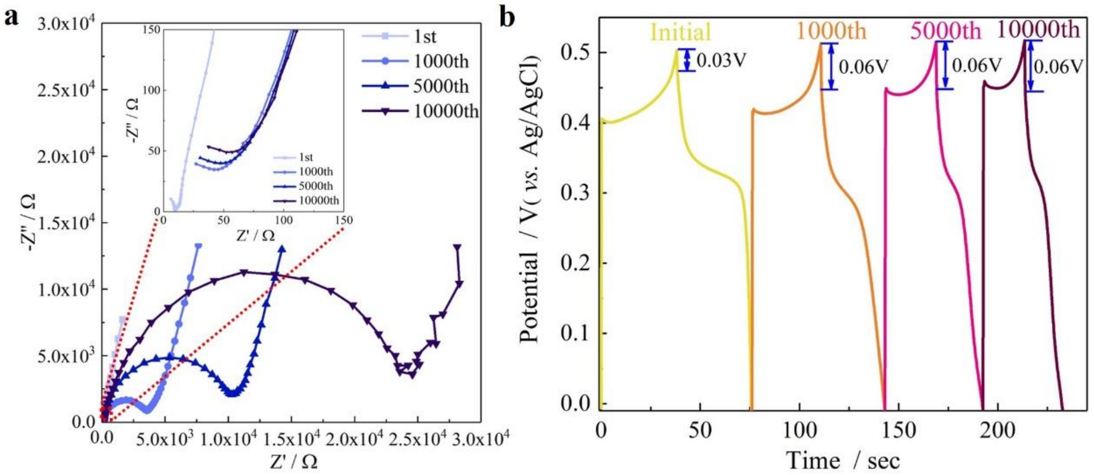

Figure S20 (a) Nyquist plots and (b) corresponding galvanostatic charge/discharge curves of the Ni-BTA nanowires film after 1, 1000, 5000 and 10000 cycles.

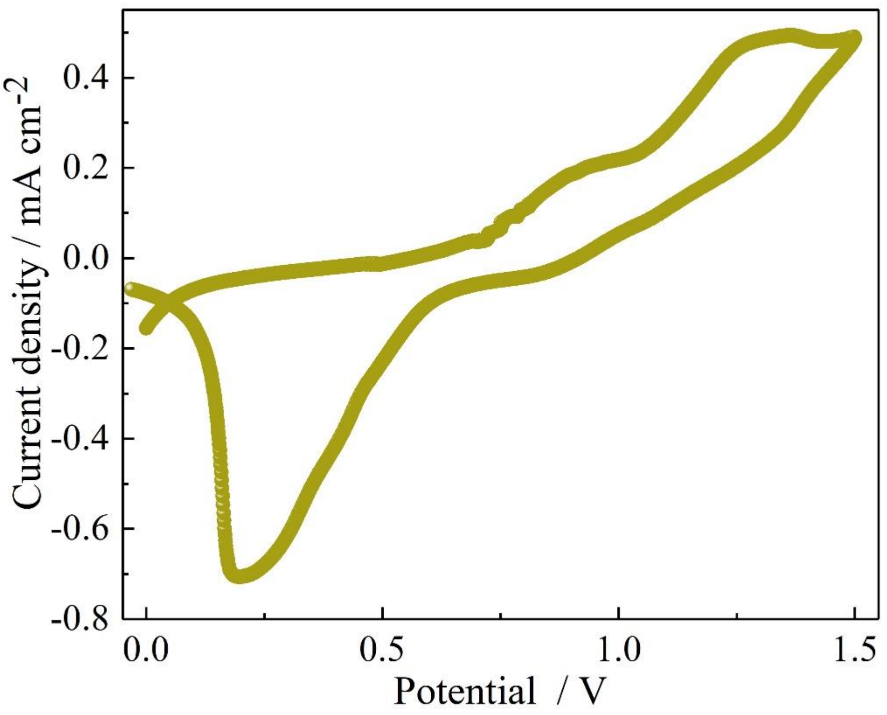

Figure S21 Cyclic voltammograms at 10 mV s^{-1} of the solid-state device was assembled by Ni-BTA nanowires film as the electrochromic layer, sprayed TiO_2 film as the ion storage layer, and KOH/ polyvinyl alcohol (PVA) as the solid electrolyte.

[s1] D.A. Keen, *J. Appl. Crystallogr.* **2001**, *34*, 172.

[s2] R. Gaillac, P. Pullumbi, K.A. Beyer, K.W. Chapman, D.A. Keen, T.D. Bennett, F.-X. Coudert, *Nat. Mater.* **2017,** *16*, 1149.

[s3] D. Van Der Spoel, E. Lindahl, B. Hess, G. Groenhof, A.E. Mark, H.J. Berendsen, *J. Comput. Chem.* **2005**, *26*, 1701.

[s4] C. Oostenbrink, A. Villa, A.E. Mark, W.F. Van Gunsteren, *J. Comput. Chem.* **2004**, *25*, 1656.

[s5] G. Kresse, J. Furthmüller, Comput. *Mater. Sci.* **1996**, *6*, 15.

[s6] G. Kresse, J. Furthmüller, *Phys. Rev. B* **1996**, *54*, 11169.

[s7] P.E. Blöchl, Projector augmented-wave method, *Phys. Rev. B* **1994**, *50*, 17953-17979.

[s8] X. Xia, Z. Ku, D. Zhou, Y. Zhong, Y. Zhang, Y. Wang, M. J. Huang, J. Tu, H. J. Fan, *Mater. Horiz.* **2016**, *3*, 588.

[s9] H. Li, L. McRae, C. J. Firby, M. Al-Hussein, A. Y. Elezzabi, *Nano Energy* **2018**, *47*, 130.

[10] G. Cai, X. Wang, M. Cui, P. Darmawan, J. Wang, A. L.-S. Eh, P. S. Lee, *Nano Energy* **2015**, *12*, 258.

[s11] D. Sheberla, J. C. Bachman, J. S. Elias, C.-J. Sun, Y. Shao-Horn, M. Dincă, *Nat. Mater.* **2016**, *16*, 220.

[s12]D. Feng, T. Lei, M. R. Lukatskaya, J. Park, Z. Huang, M. Lee, L. Shaw, S. Chen, A. A. Yakovenko, A. Kulkarni, J. Xiao, K. Fredrickson, J. B. Tok, X. Zou, Y. Cui, Z. Bao, *Nat. Energy* **2018**, *3*, 30.
